# Bilateral House Flap Anoplasty for Severe Anal Stenosis: Our Experience at a Tertiary Care Center in India

**DOI:** 10.7759/cureus.88709

**Published:** 2025-07-24

**Authors:** Vasu Vashishtha, Brij B Agarwal, Ayush K Mishra, Neeraj Dhamija

**Affiliations:** 1 General and Laparoscopic Surgery, Sir Ganga Ram Hospital, New Delhi, IND

**Keywords:** anal pain, anal stenosis, bilateral house flap anoplasty, chemical irritant, constipation

## Abstract

Anal stenosis is a narrowing of the anal canal that can result from either an anatomic stricture or functional obstruction. While postoperative and idiopathic causes are more common, chemically induced anal stenosis following the application of traditional medicine for hemorrhoids is rare and often severe. Literature on this etiology and its management is scarce. This case highlights the need for health education and raises awareness among general surgeons who may encounter such cases, particularly in regions where traditional medicine is widely used.

We report the case of a 38-year-old gentleman from the state of Bihar who presented with a three-month history of progressive difficulty in passing feces and flatus, requiring digital evacuation. These symptoms were also accompanied by intermittent abdominal distension. Notably, he had undergone traditional medicine application for hemorrhoidal treatment four months prior, which initially resulted in ulceration and subsequently healed with scarring. He had no history of diabetes, smoking, or other chronic illnesses.

Physical examination confirmed severe anal stenosis. The patient underwent bilateral House advancement flap anoplasty, performed by a general surgeon, with excellent functional outcomes.

The most common cause of anal stenosis is hemorrhoidectomy, followed by other anorectal surgeries, anorectal diseases, and radiotherapy. Diagnosis is primarily clinical. While mild cases can be managed conservatively, moderate to severe cases require surgical intervention, with advancement flap anoplasty being a preferred approach.

Health education is essential to prevent complications arising from inappropriate treatments. House advancement flap anoplasty is a viable and effective surgical option for anal stenosis in resource-limited settings due to its technical simplicity and favorable outcomes.

## Introduction

Anal stenosis is a pathological narrowing of the anal canal, resulting from either true anatomic stricture or functional hypertonicity of the internal anal sphincter. Anatomic stenosis occurs when the normal anoderm is replaced by fibrotic scar tissue, most commonly following hemorrhoidectomy, which accounts for approximately 90% of cases. A less common but severe cause is chemical-induced anal stenosis, resulting from the application of irritant substances by non-medical practitioners. These corrosive agents cause mucosal burns that heal with fibrosis, leading to progressive stenosis. Despite its clinical significance, chemical-induced anal stenosis is rarely reported in the literature. In resource-limited settings, a shortage of trained healthcare professionals and the widespread reliance on traditional healers contribute to its prevalence [1,2]. A 2003 report documented 24 cases of anal stenosis, with seven attributed to chemical application by non-medical practitioners, five of which resulted in severe stenosis. Compared to other etiologies, chemically induced stenosis tends to be more severe and often necessitates complex surgical intervention [3]. Cases of complete anal canal obliteration following prolonged exposure to corrosive substances have also been documented, leading to significant morbidity, including chronic pain, abdominal distension, and refractory constipation.

Diagnosis is primarily clinical, with severity classified as mild, moderate, or severe based on digital examination or assessment with a Hill-Ferguson retractor [4]. Common symptoms include painful defecation, constipation, reduced stool caliber, obstipation, and rectal bleeding. While mild stenosis may be managed conservatively, moderate to severe cases typically require surgical intervention. Treatment techniques generally aim to increase the anal canal size by use of local skin flaps, for example, triangular (Y-V) or square-shaped sliding grafts; however, tension or ischemia can cause necrosis at the terminal portion of the skin flap [5]. House advancement flap anoplasty is relatively easy to perform and was developed to avoid the limitations of other flap procedures while preserving their benefits [6].

## Case presentation

A gentleman, 38 years of age from the state of Bihar in India, presented with a three-month history of progressive difficulty in passing feces and flatus, eventually requiring digital evacuation of feces. This was also accompanied by intermittent abdominal distension. Notably, he had undergone traditional medicine application for hemorrhoidal treatment 4 months prior, which initially resulted in ulceration that subsequently healed with scarring. He had no history of diabetes, smoking, or other chronic illnesses. On examination, his general physical, abdominal, and inguinoscrotal examinations were normal. Perineal examination revealed severe anal stenosis with a circumferential thick scar, preventing even the insertion of the examiner’s little finger. Routine laboratory investigations were within normal limits. Based on clinical findings, a diagnosis of severe anal stenosis was established, and the patient was scheduled for surgical intervention after obtaining consent and explaining the expected postoperative outcomes.

Surgical procedure

**Figure 1:**
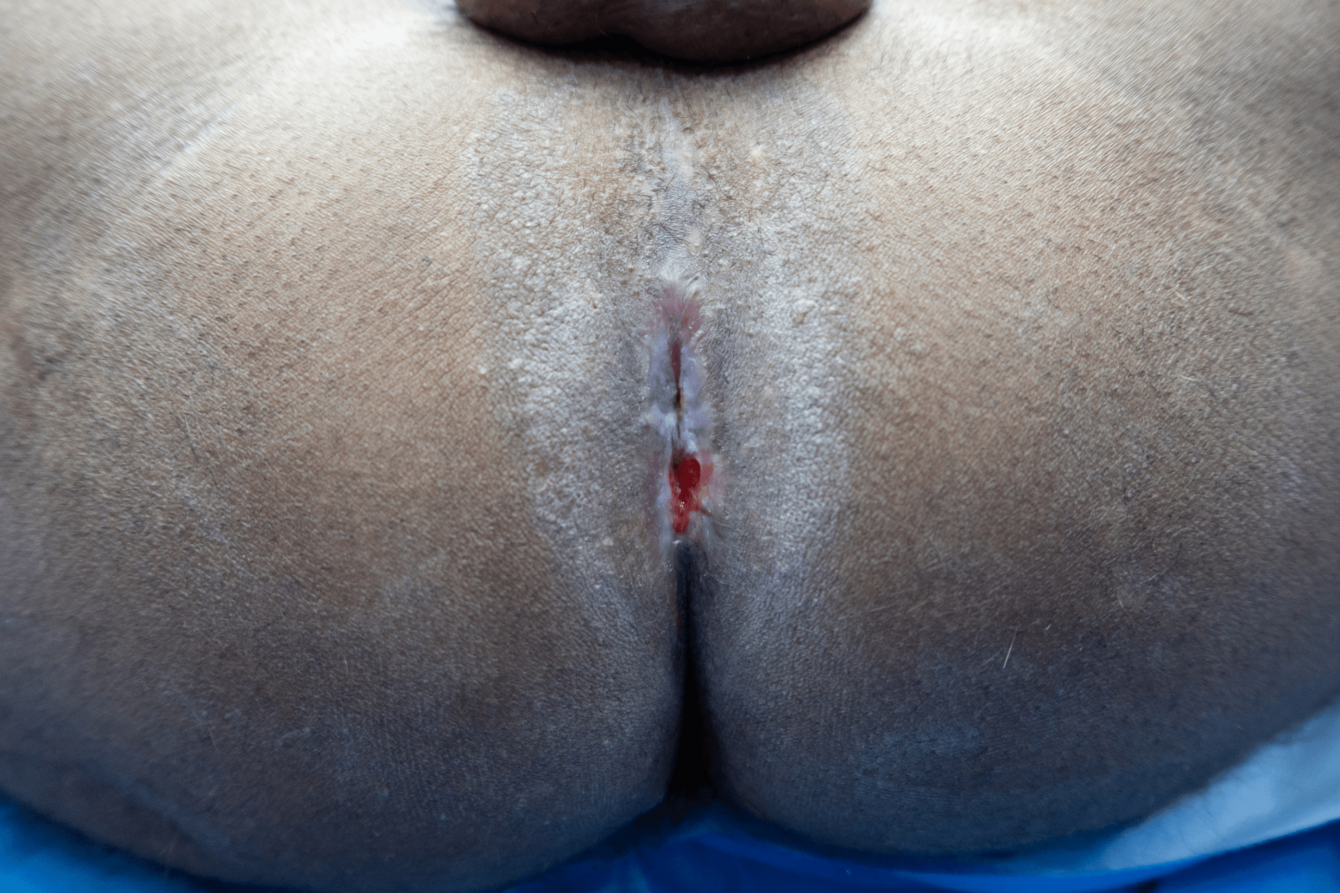
Stenosed anal canal with surrounding cicatricial tissue.

**Figure 2:**
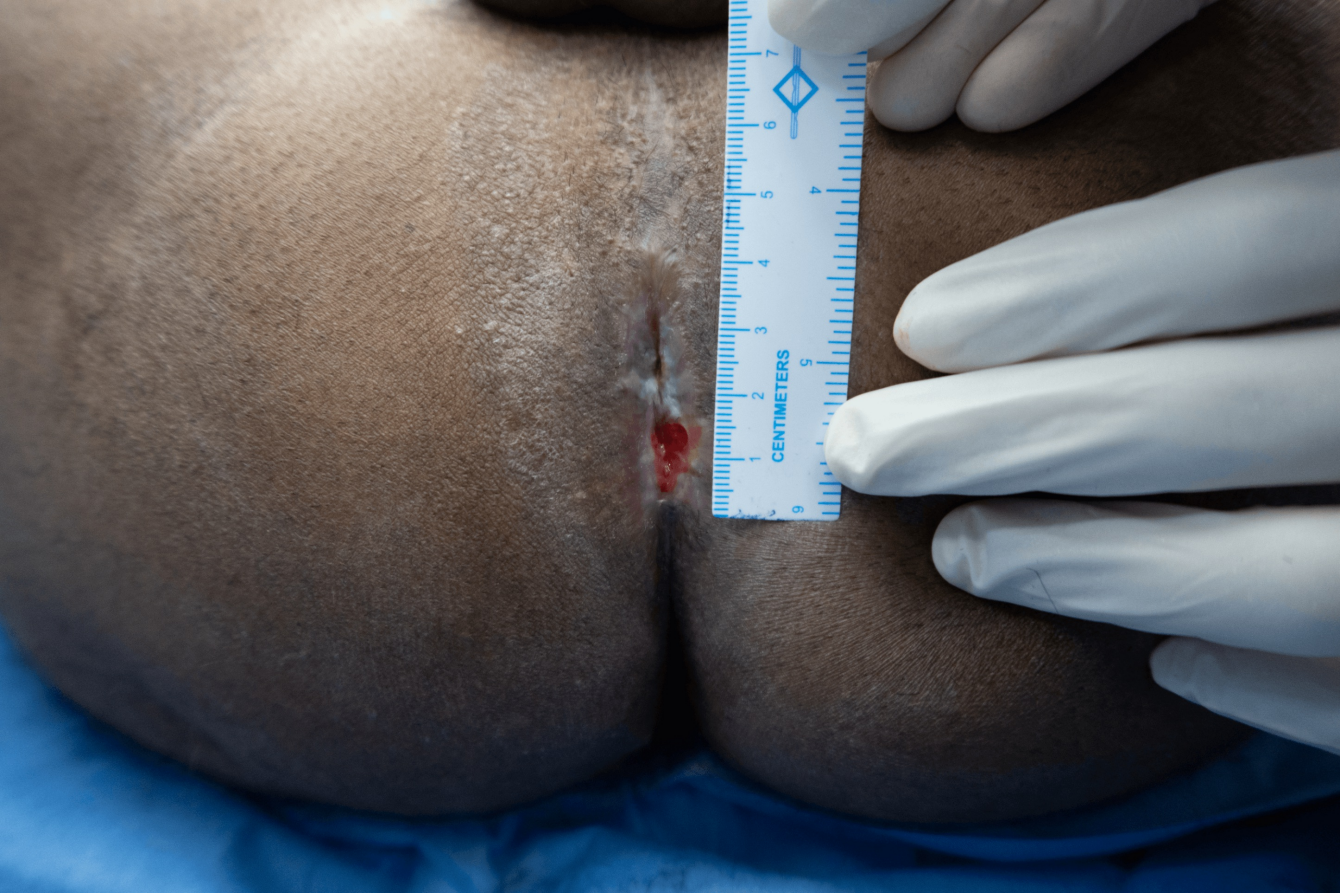
Preoperative measurement showing a 1 cm diameter of the anal opening.

**Figure 3:**
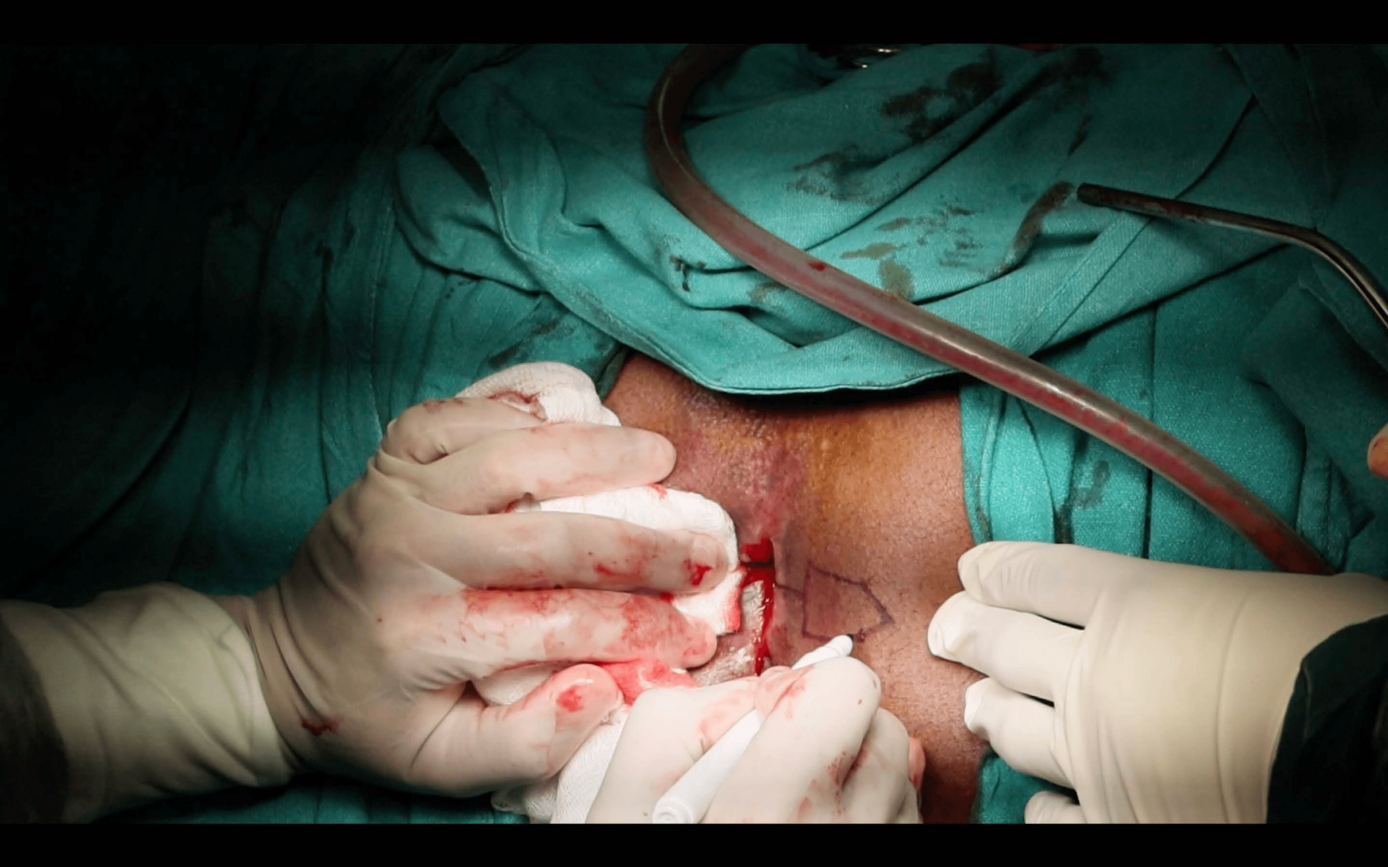
“House”-shaped markings for flaps made on both sides.

Preoperatively, IV third-generation cephalosporin and aminoglycoside were administered and continued postoperatively. Under general anesthesia, the patient was placed in the lithotomy position, painted, and draped following standard aseptic protocols. Examination findings were confirmed, the anal canal was severely stenosed, not allowing even the examiner’s little finger, as seen in Figure 1. Pre-operative marking of the anal opening was done, which measured 1 cm in diameter, as shown in Figure 2. Marking of the incision and proposed flap was then performed (Figure 3).

**Figure 4:**
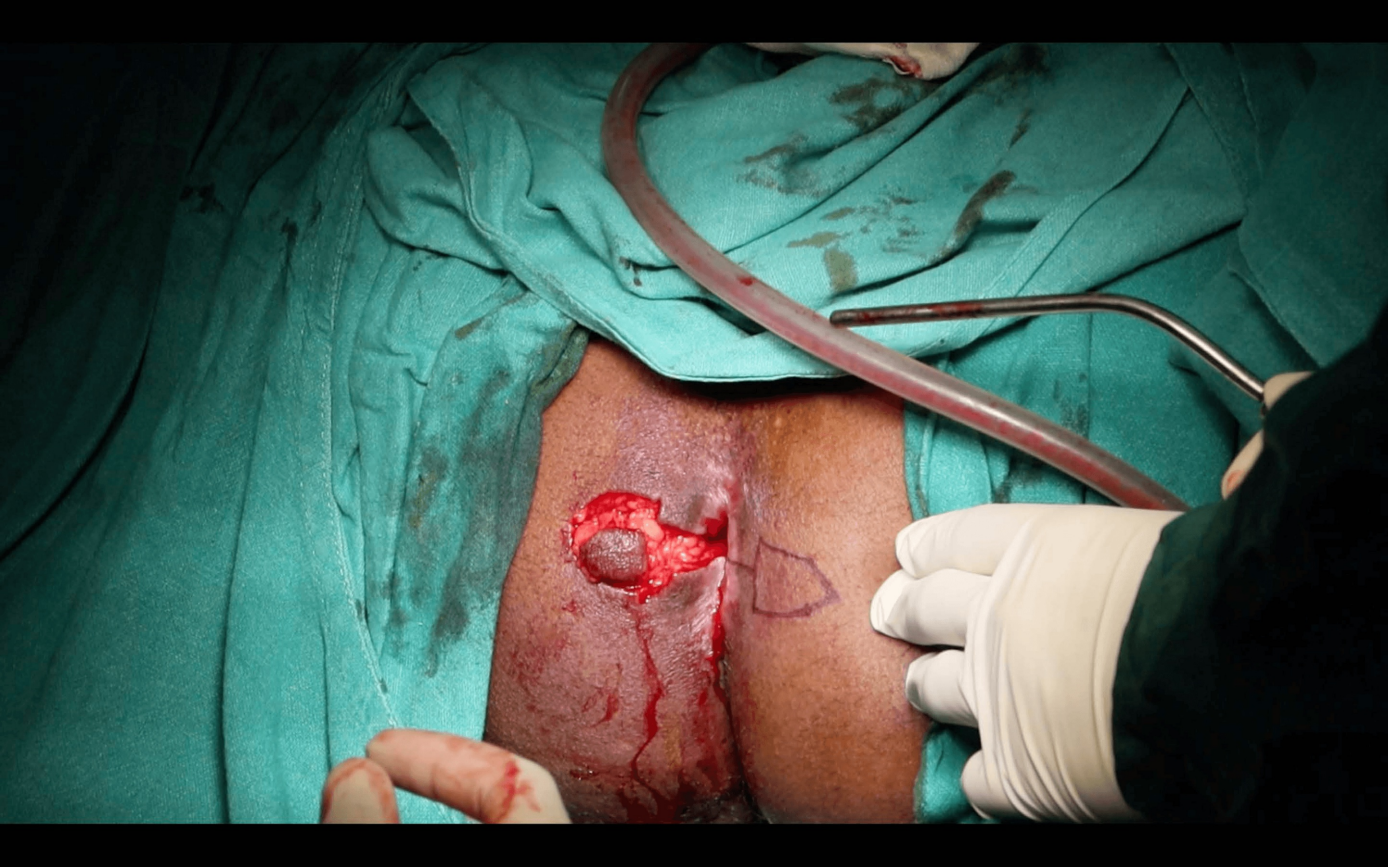
Horizontal incision made and House-shaped flap mobilized.

**Figure 5:**
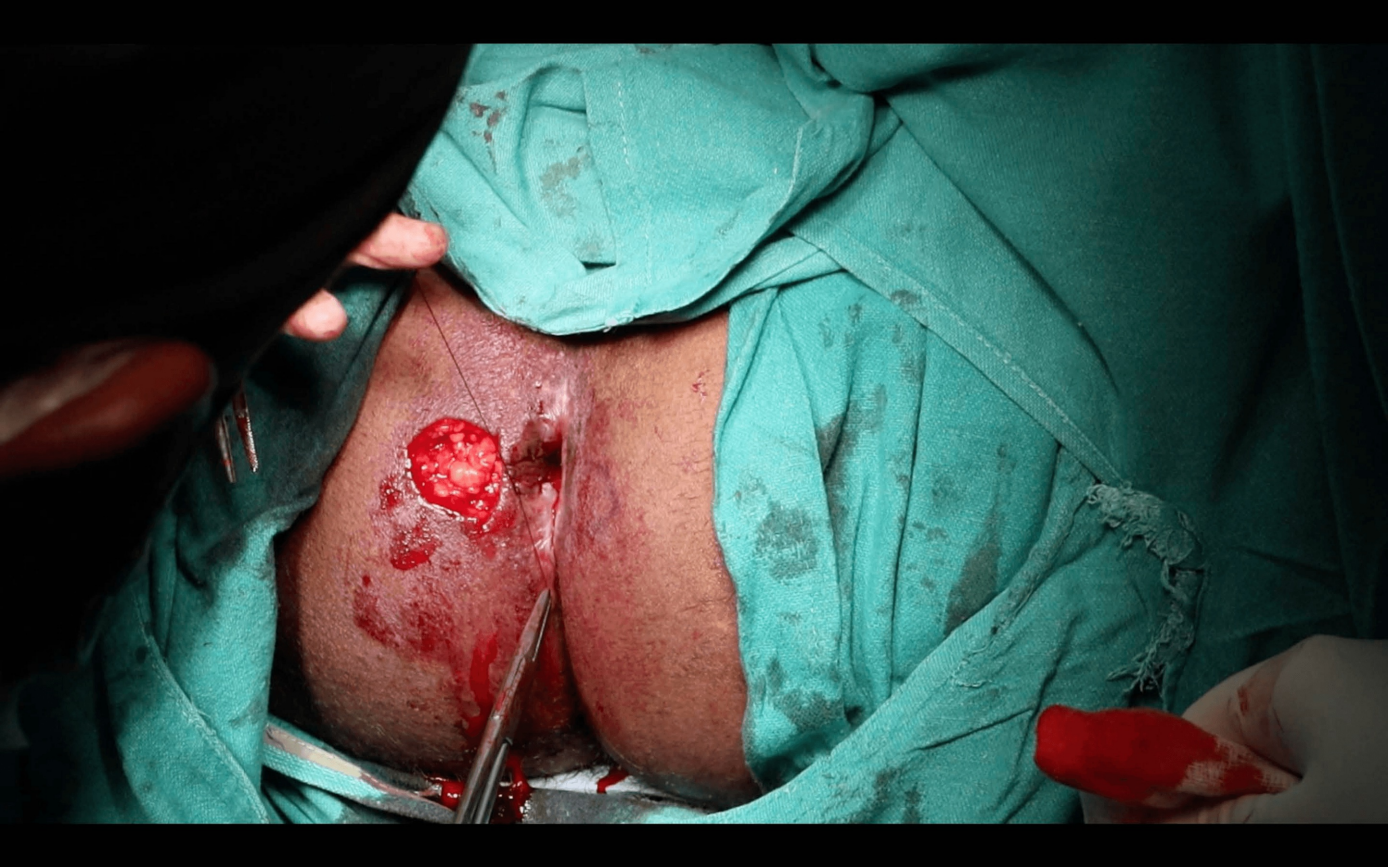
Suturing of the base of the flap with the mobilized epithelial component.

A horizontal incision was made at the 9 o’clock position, extending from the distal to the proximal extent of the circumferential scar, approximately 2-3 mm distal to the dentate line. A pentagon-shaped House advancement flap was designed, ensuring the flap length was twice its base width. The scar tissue was partially excised both anteriorly and posteriorly to the initial incision, and the flap was meticulously developed to preserve vascular integrity (Figure 4). The flap base was sutured to the rectal mucosa using 2-0 absorbable polyglactin 910 sutures, while its lateral edges were secured to the anoderm and perineal skin (Figure 5).

**Figure 6:**
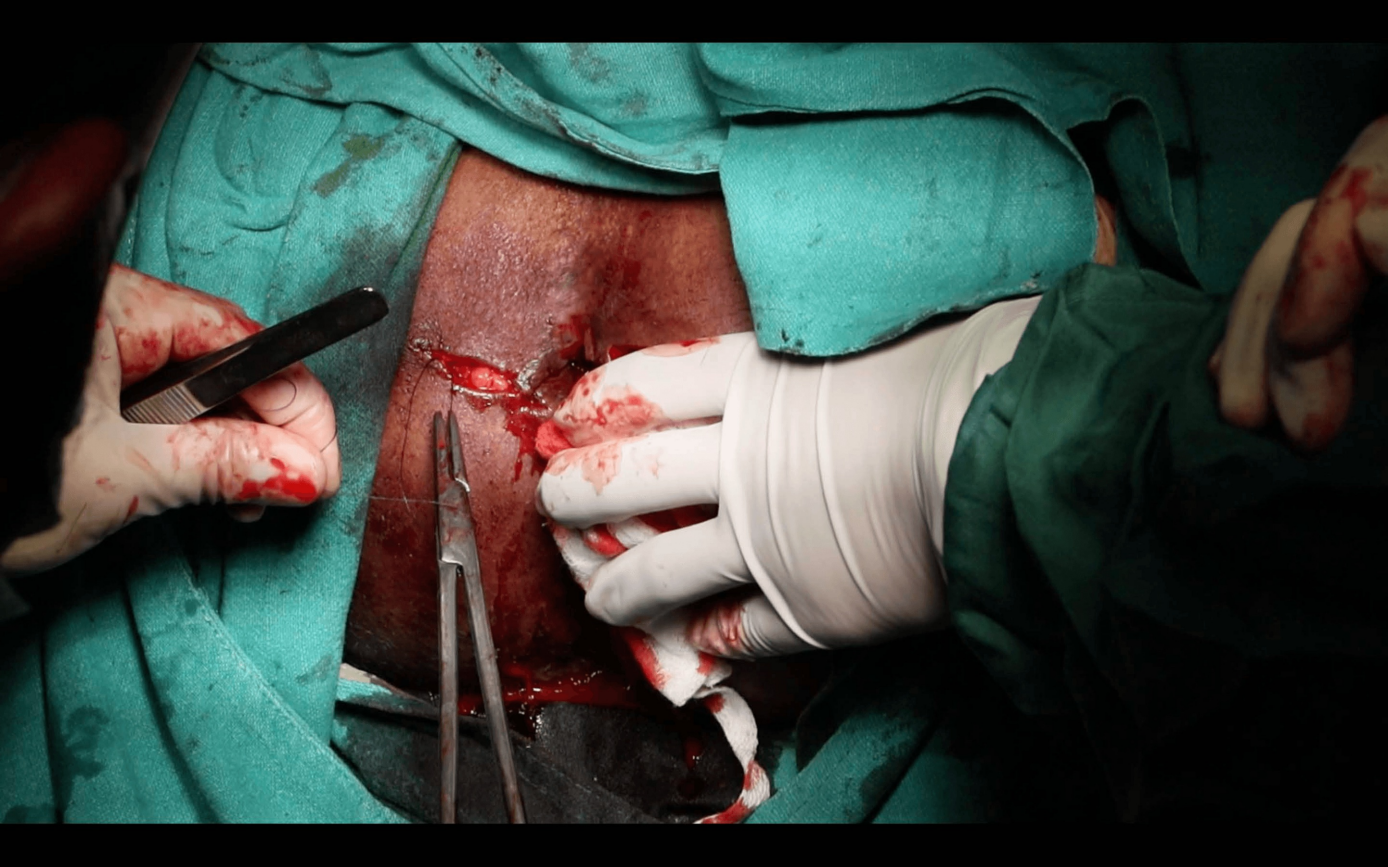
Closure of the incision beyond the apex of the flap.

**Figure 7:**
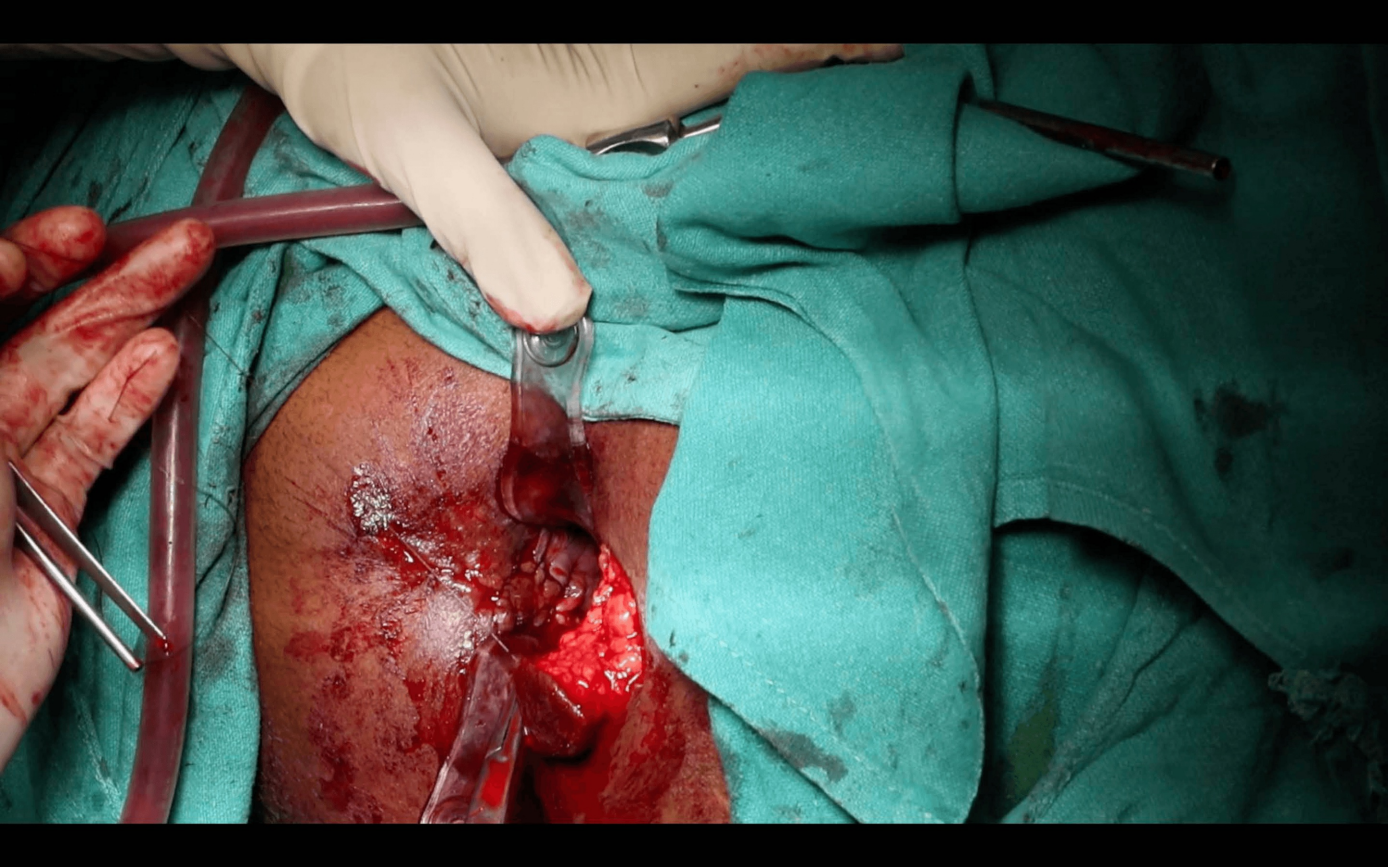
Similar flap raised on the opposite side.

**Figure 8:**
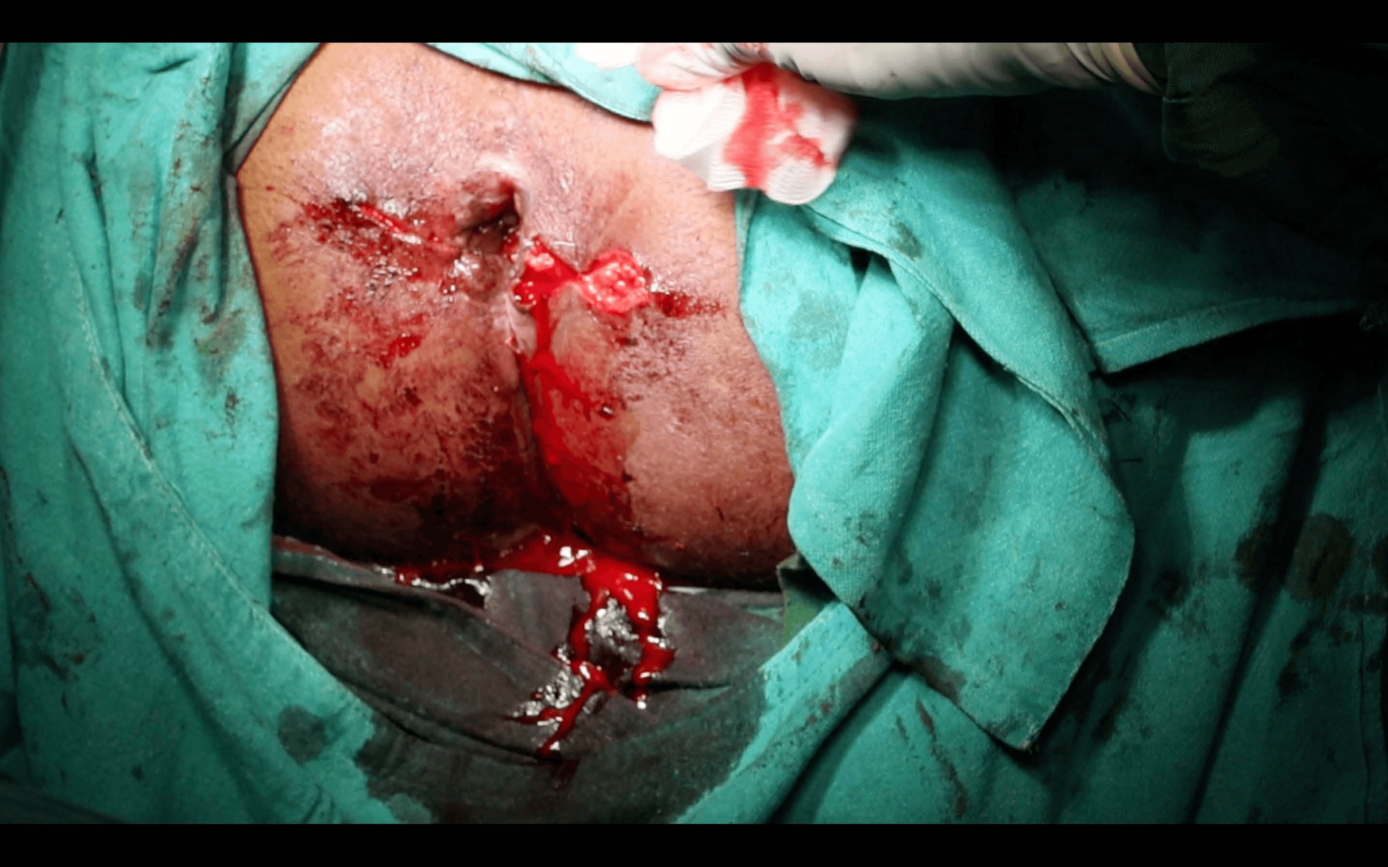
Both sides sutured symmetrically.

**Figure 9:**
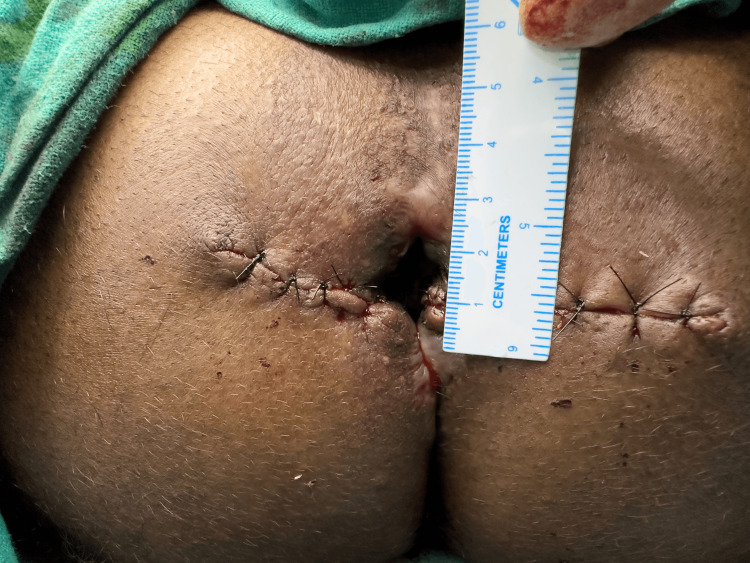
Postoperative image showing increased anal opening (2.5 cm).

**Figure 10:**
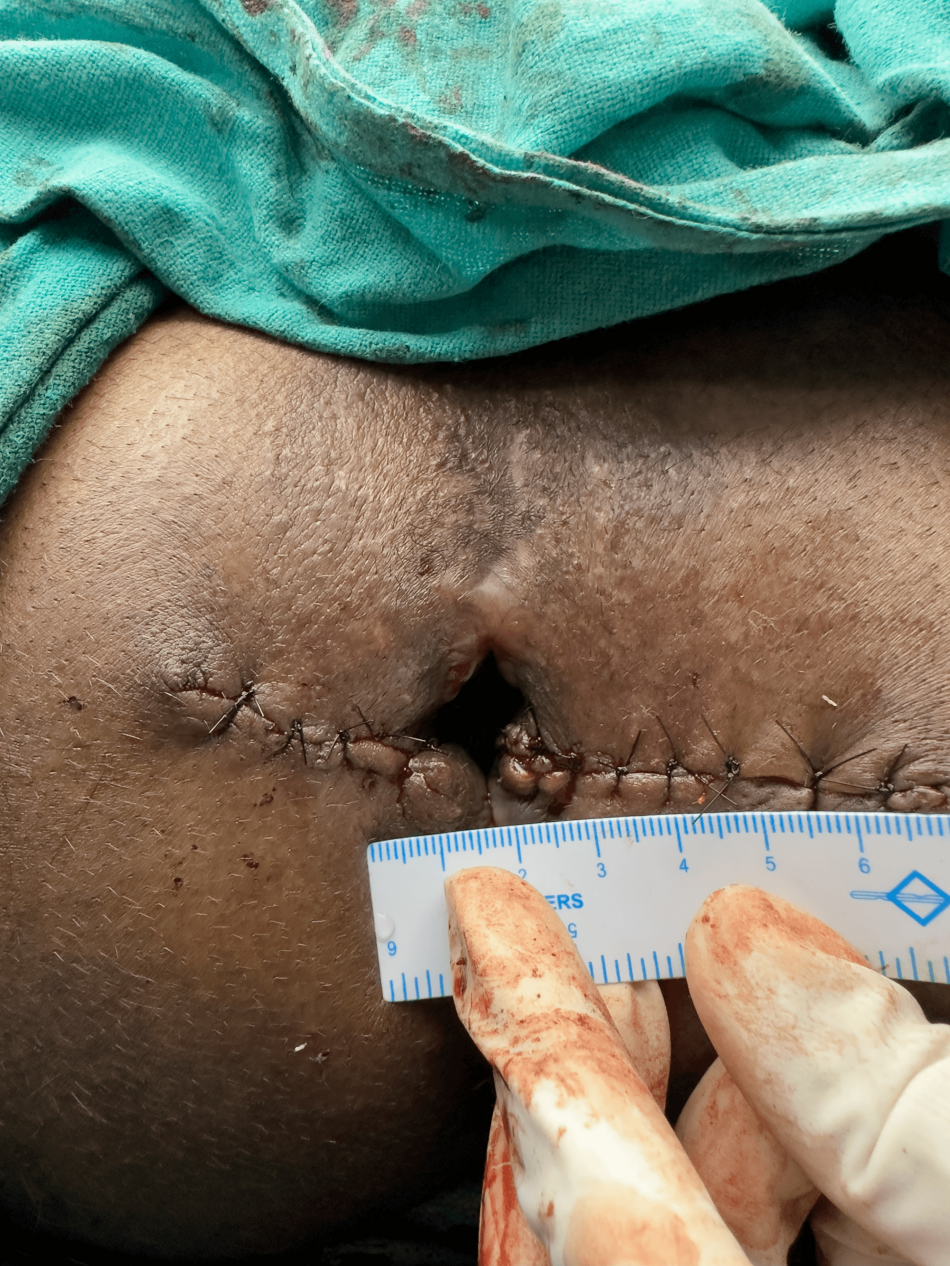
Transverse diameter measuring approximately 2.5 cm.

**Figure 11:**
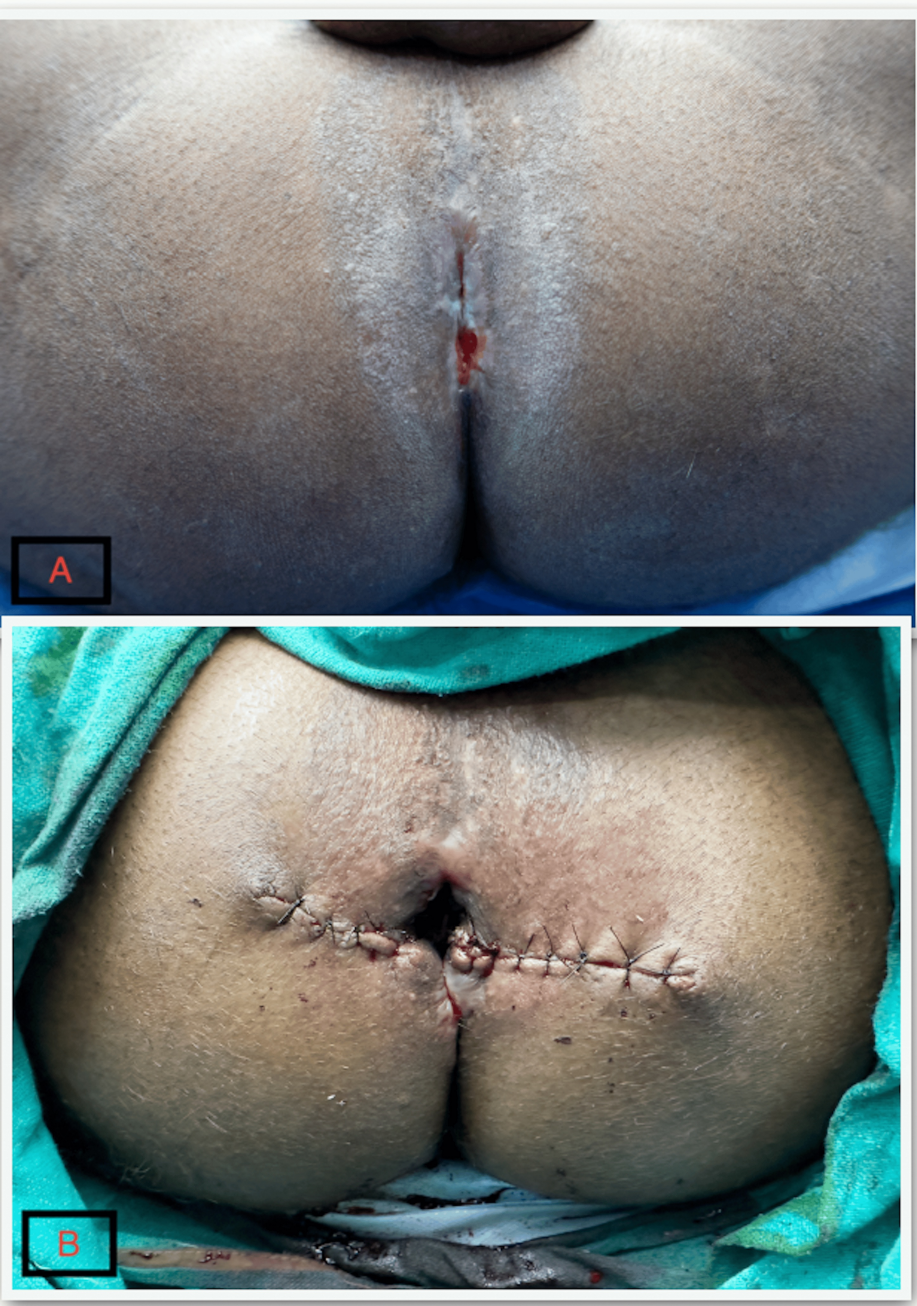
Side-by-side comparison of preoperative and postoperative anal opening. 11A: Preoperative anal opening (anal stenosis); 11B: Postoperative anal opening.

The distal wound beyond the apex of the flap was closed primarily using 3-0 absorbable polyglactin 910 (Figure 6). The procedure was then symmetrically performed on the contralateral side by making a horizontal incision and raising a House flap (Figure 7). As seen in Figure 8, horizontal incisions on both sides were symmetrically closed. Skin closure of incisions beyond the apex on both sides was done using 3-0 nylon sutures. The diameter of the anal opening increased to 2.5 cm (Figures 9-10). A significant increase in the size of the anal opening was noted post-operatively (Figure 11B) compared to the pre-operative diameter (Figure 11A).

The patient was discharged on oral amoxicillin-clavulanic acid for five days, along with stool softeners and sitz baths starting from the second postoperative day until complete wound healing.

Outcome and follow-up 

**Figure 12:**
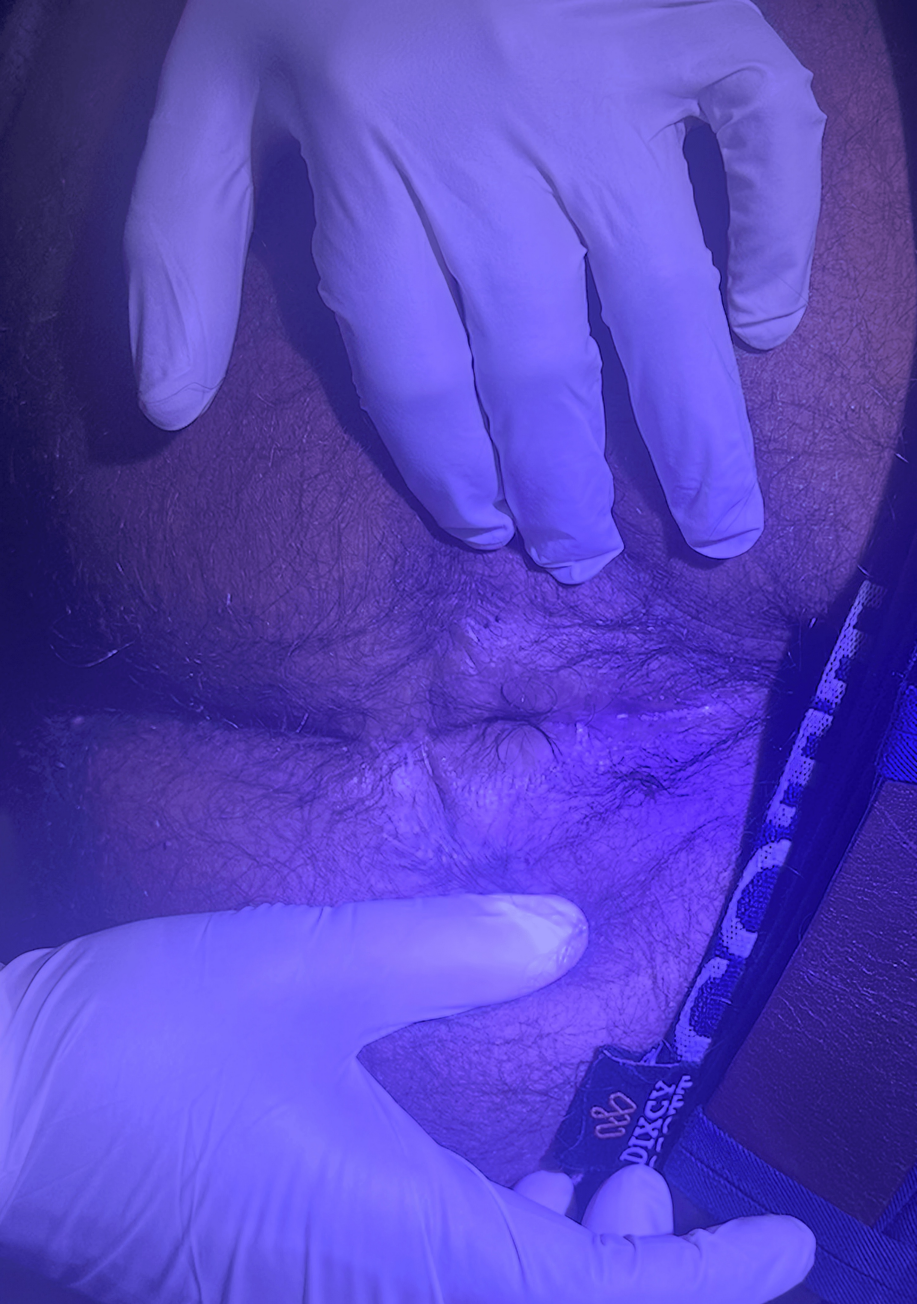
Well-healed wound with improved anal opening after 1 year.

The patient followed up in the OPD with well-healed suture sites. The anal opening had increased to 2.5 cm, with some straining on defecation after 2 weeks, no further requirement for digital evacuation, and preserved continence. He was advised to undergo progressive anal dilatation using anal dilators. Following anal dilation, the parameters mentioned above improved further. At one year of follow-up, the anal opening measured 3.5 cm with a normal anal tone (Figure 12).

## Discussion

Various surgical techniques have been employed to restore normal anatomy and function in patients with anal stenosis. While complex procedures such as S-plasty were previously utilized, they have largely fallen out of favor due to high morbidity and prolonged hospital stays. However, these techniques may still be considered in cases of severe high strictures associated with mucosal ectropion, failed prior interventions, or extensive skin excision requiring tissue rotation, such as in the surgical management of Paget’s disease [6-9].

For most patients, relatively simple reconstructive techniques such as Y-V anoplasty, rhomboid flaps, and House advancement flaps are preferred as first-line surgical options. Several observational and retrospective studies have evaluated the outcomes of Y-V anoplasty and various advancement flap techniques. Angelchik PD et al. reported on 19 patients treated with Y-V anoplasty or a diamond-shaped pedicle advancement flap for anal stenosis or ectropion. They observed complications in three patients following Y-V anoplasty and suggested that the risk of contraction, infection, and flap necrosis may be lower with the diamond flap technique [8].

In a study by Aitola PT et al., nine out of ten patients showed postoperative improvement, with six achieving good results, three experiencing fair outcomes, and one having a poor result. Similarly, Maria G et al. evaluated 42 patients with severe anal stricture, of whom 29 underwent Y-V anoplasty and 13 received a diamond flap anoplasty. Among those treated with Y-V anoplasty, early postoperative complications included suture dehiscence in three patients, ischemic contracture of the leading edge of the flap in one patient, and urinary tract infections in two patients. At a two-year follow-up, 93% of patients were deemed successfully treated, while 7% (all of whom underwent Y-V anoplasty) showed improvement but did not achieve full resolution. The overall complication rate for Y-V anoplasty was 15%. The authors concluded that while both Y-V and diamond flap anoplasty were effective, the diamond flap technique was associated with reduced suture line tension, improved vascular supply, and greater reliability [10].

Casadesus D et al. conducted a retrospective review of 19 patients who underwent surgical management for moderate to severe anal stenosis, further supporting the efficacy of these reconstructive approaches [11].

Christensen MA et al. were the first to describe the House advancement pedicle flap for the treatment of anal stenosis, highlighting several advantages of the technique. These include providing a broad skin flap to cover the entire length of the affected anal canal, enabling primary closure of the donor site, and avoiding extensive tissue mobilization. Additionally, the procedure maintains adequate vascular supply with minimal tension and eliminates the risk of necrosis-prone small flap tips. Unlike Y-V and mucosal advancement flaps, which rely on a skin or mucosal bridge for perfusion, the House flap derives its blood supply from vessels traversing a fatty pedicle originating from the underlying external sphincter muscle [12].

Sentovich SM et al. reported significant improvement in 26 of 29 patients (median follow-up: 28 months) after undergoing House flap anoplasty [13].

Similarly, Alver O et al. evaluated 28 patients who underwent the procedure for various anorectal conditions, including eight cases of anal stenosis. Apart from one patient with a rectovaginal fistula who experienced recurrence, all patients were satisfied with the outcome. Minimal wound dehiscence occurred in three cases, but no instances of flap necrosis or recurrent stenosis were observed. All eight patients treated for anal stenosis achieved favorable outcomes, further supporting the efficacy of the House advancement flap in managing this condition [6].

Farid M et al. conducted a prospective randomised study comparing the “House flap,” “Y-V anoplasty,” and “Diamond flap” for anal stenosis. They divided 60 patients randomly into three groups undergoing the three different procedures. They found that the “House advancement flap” had better clinical outcomes and greater improvement in quality of life (measured using GIQLI), with the only disadvantage being a longer operative time [14].

We performed the House advancement flap anoplasty bilaterally for our patient, who presented with a moderate to severe grade of anal stenosis. The patient was also advised to use progressive anal dilators following satisfactory wound healing. The quality of life significantly improved following the procedure, although no objective assessment was performed. It becomes essential to choose the appropriate surgical procedure in the setting of anal stenosis developing as a consequence of malpractices and quackery to ensure the best results. In South Asian countries with a growing incidence of quackery and malpractices, prevention through public education, as well as treatment of anal stenosis with a ‘standard of care’ procedure like the House advancement flap, is the need of the hour. To our knowledge, no such procedure has been reported in the Indian subcontinent based on our literature search.

## Conclusions

Anal stenosis, particularly in developing countries, can result from the application of toxic substances by traditional healers in the treatment of hemorrhoids. In settings with limited access to colorectal surgeons, experienced general surgeons can effectively manage this condition. The House advancement flap remains a reliable surgical option, offering technical simplicity and favorable outcomes in terms of symptom relief and quality of life. Strengthening public health education is essential to discourage patients from seeking unsafe treatments, while engaging traditional healers through scientific collaboration may help reduce the use of harmful practices.
